# Case report of chondroma in a grass carp (*Ctenopharyngodon idella*)

**Published:** 2016-06-15

**Authors:** Mehrzad Mesbah, Annahita Rezaie, Zahra Tulaby Dezfuly

**Affiliations:** 1 *Department of Clinical Sciences, Faculty of Veterinary Medicine, Shahid Chamran University of Ahvaz, Ahvaz, Iran; *; 2 *Department of Pathobiology, Faculty of Veterinary Medicine, Shahid Chamran University of Ahvaz, Ahvaz, Iran; *; 3 *PhD student of Aquatic Animal Health, Department of Clinical Sciences, Faculty of Veterinary Medicine, Shahid Chamran University of Ahvaz, Ahvaz, Iran.*

**Keywords:** Chondroma, Histopathology, Grass carp

## Abstract

The grass carp (*Ctenopharyngodon idella*) is a herbivorous, freshwater fish species of the family Cyprinidae, and the only species of the genus *Ctenopharyngodon*. Neoplasms in ﬁshes are generally less aggressive than neoplasms in mammals and are most commonly discrete, focal and benign neoplasms. A 3-year-old grass carp with a big mass on the vertebrae was referred to the clinic. According to the owner’s statements, the fish had no signs of lethargy, ataxia and abnormal behaviors. The size of the mass was 7 × 6 × 6 cm. It cut hardly with audible sounds. The consistency of the mass was as hard as a cartilage. Microscopic examination revealed numerous irregular crests of hyaline cartilage beneath the skin. According to histopathologic characteristics, chondroma on the vertebrae of grass carp was diagnosed.

## Introduction

The grass carp (*Ctenopharyngodon idella*) is a herbi-vorous, freshwater fish species of the family Cyprinidae, and the only species of the genus *Ctenopharyngodon*. It is a large cyprind native to eastern Asia with a native range from northern Vietnam to the Amur River on the Siberia-China border. Grass carp is a sub-tropical to temperate species. Grass carp have been extensively introduced, mainly for macrophyte control, to many parts of the world.^1^ Spontaneous neoplasms of cartilage have been reported and include chondromas of the gill arch or ﬁlamental cartilage,^2^ osteochondroma in the jewel ﬁsh (*Hemichromis bimaculatus*),^3^ and chondrosarcomas of the paddleﬁsh^4^ and mangrove rivulus.^5^ A branchial osteochondroma has also been reported in a gilthead sea bream.^6^ The aim of the present paper was description of chondroma in a grass carp and to the best of author’s knowledge this is the first report.

## Case Description

A 3-year-old grass carp had a big mass on the vertebrae. According to the owner’s statements, the fish had no signs of lethargy, ataxia, and abnormal behavior. During fishing a big mass had been seen on the fish vertebrae and then it was referred to the veterinary teaching hospital. The other fish were normal. After macro-scopic examination the mass was dissected out and fixed in formalin buffer 10%. Routine procedures of pathology laboratory were performed and semi-thin sections were stained with hematoxylin and eosin and periodic acid–Schiff (PAS). Then they were assessed under light microscope.

## Results

**Fig. 1:**
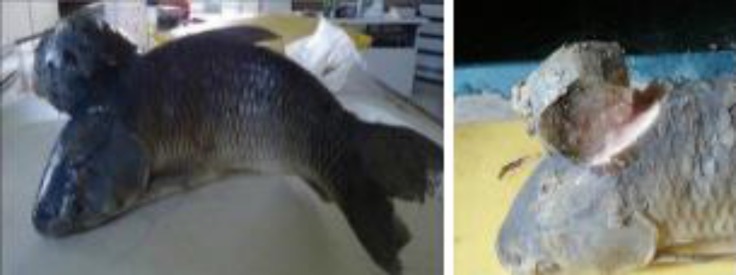
Chondroma. Note the big mass on the vertebrae of grass carp and transverse section of the tumor

**Fig. 2:**
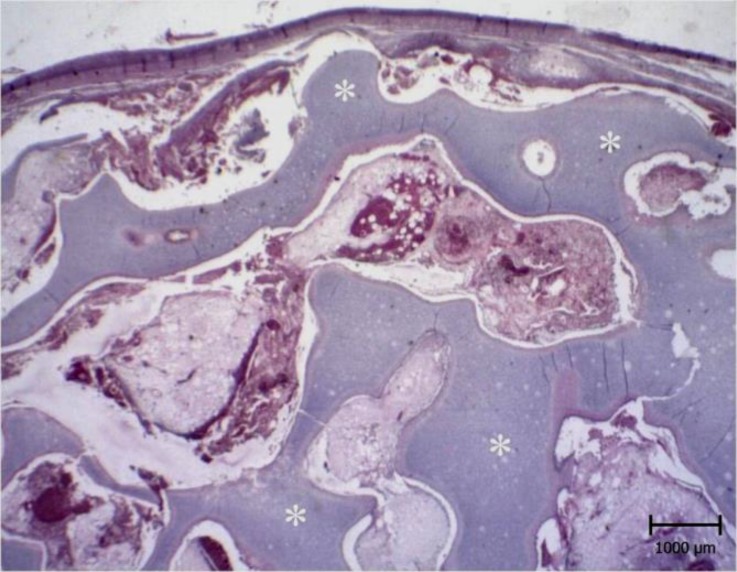
Chondroma. Note the numerous irregular crests of hyaline cartilage beneath the skin (white stars), (H & E).

**Fig. 3:**
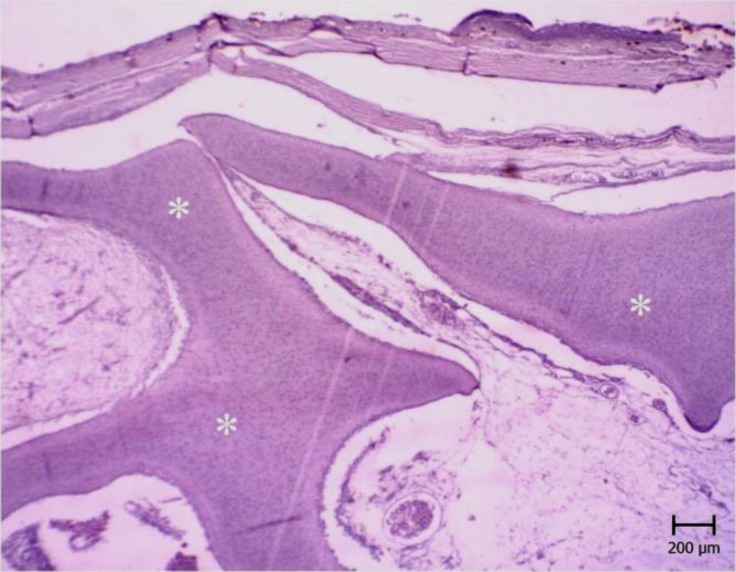
Chondroma. Note the irregular crests of hyaline cartilage (white stars), (PAS).

**Fig. 4:**
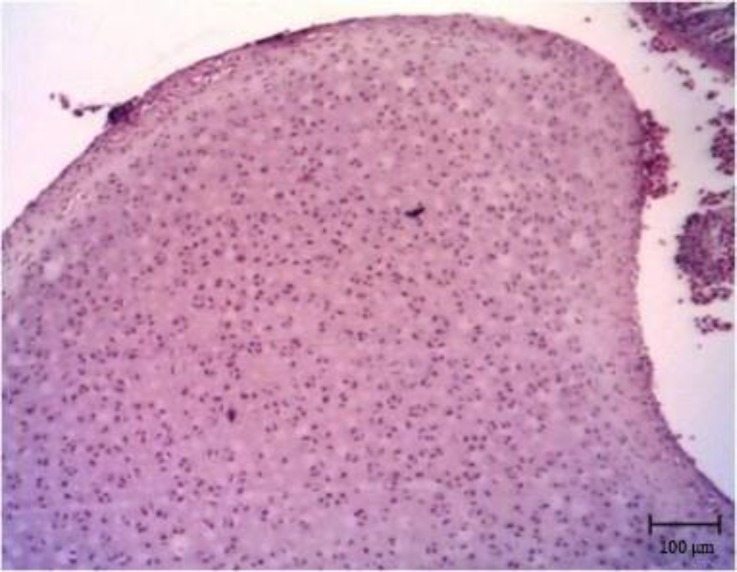
Chondroma. Note thr cartilage structure with lacunae and chondrocytes (H & E).

The size of the mass was 7 × 6 × 6 cm. It cut hardly with audible sounds. The consistency of the mass was as hard as a cartilage (Fig. 1). The cross section was white to pink and there were many cavernous structures which were filled with brownish fluid. Microscopic examinations revealed numerous irregular crests of hyaline cartilage beneath the skin. The crests had different thicknesses (Figs. 2 and 3). Chondroblasts were laid on outer layer of crests. They were cuboidal with scant cytoplasm (Fig. 4).

**Fig. 5:**
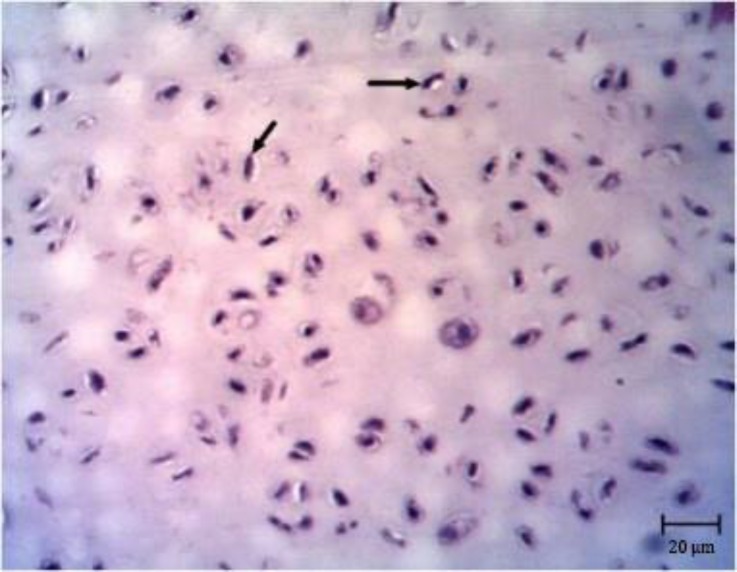
Chondroma. Note the chondrocyte cells located in lacuna (black arrows), (H & E

**Fig. 6:**
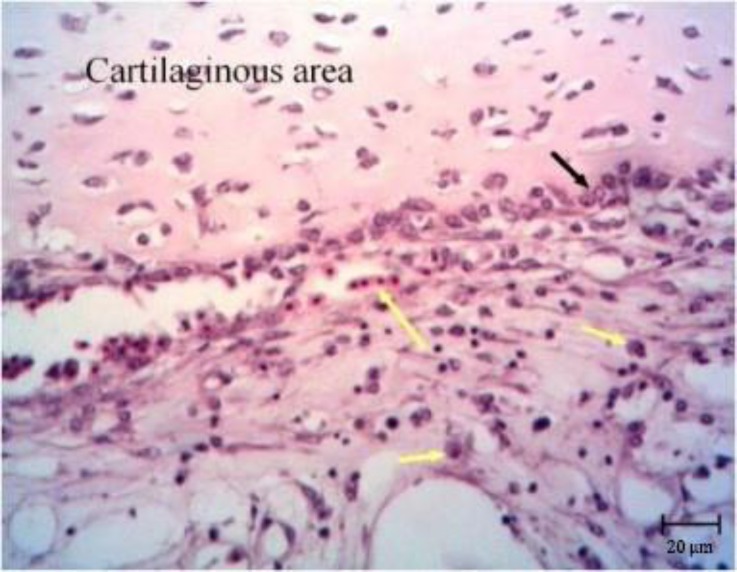
Chondroma. Note the chondroblasts laid on outer layer of crests (black arrow) and inflammatory cells (yellow arrows), (H & E)

Chondrocyte cells were located in lacuna and isogenic groups were common (Fig. 5). They were suited in basophilic matrix of cartilage. There were spaces between crests of cartilage. In several cavities, numerous blood vessels and inflammatory cells were seen (Fig. 6). These cells were often neutrophils.

## Discussion

Fish neoplasms are generally less aggressive than neo-plasms in mammals and are the most commonly discrete, focal and benign neoplasms.^2^ Development of neoplasm in fish is a multifactorial and multistage process that may result from acquired genetic characteristics^7^ or exposure to various environmental agents such as chemicals^8^ or infectious agents including viral agents^9^ as in other vertebrates. Although the etiology of majority of fish spontaneous neoplasm has not been determined, however, genetic predisposition is inherently an important factor in development of neoplastic disease in all species including ﬁsh.^10^

Diagnostic procedures for diﬀerentiation of neoplastic lesions from non-neoplastic lesions in ﬁsh are generally limited to wet-mount, cytological, histological, and ultra-structural examinations of the lesions. Direct wet-mount examination of any lesion is a simple, however, a highly diagnostic procedure that should be included in the routine diagnostic protocol of the aquatic animal practitioner.^11^ In this case histopathologic examination could detect the entity of the mass and it is completely reliable.

The treatment of neoplastic disease in ﬁsh is generally restricted to surgical intervention, whereas other treatment modalities used in mammalian species such as radiation and chemical therapies are not practical in ﬁsh.^12^

 Neoplasm in several groups of ﬁsh such as chondrichthyan (class chondrichthyes), elasmobranchs (sharks, skates, and rays),^13^ holocephalans (chimeras) and other primitive fish including African lungﬁsh (*Protopterus* spp.; family Protopteridae)^14^, bowﬁn (Family Amiidae)^15^ and the chondrostean fish (sturgeons and paddleﬁshes; Order Acipensiformes)^4^ have been reported. Intrinsic factors such as age, gender and genetic predisposition and extrinsic factors such as temperature, season and environmental quality are factors related to the occurrence of neoplastic disease in ﬁsh.^16^

According to histopathologic characteristics, chondroma on vertebrae of grass carp was diagnosed. To the best of authors’ knowledge this case appeared to be the first report of chondroma in *Ctenopharyngodon idella*.
